# Risk factors associated with locoregional failure and estimation of survival after curative resection for patients with distal bile duct cancer

**DOI:** 10.1038/s41598-019-41622-2

**Published:** 2019-03-25

**Authors:** Jung Ho Im, Joon Seong Park, Dong Sup Yoon, Dong Ki Lee, Jun Won Kim, Ik Jae Lee

**Affiliations:** 1 Department of Radiation Oncology, CHA Bundang Medical Center, CHA University School of Medicine, Seongnam, Korea; 2 Pancreatobiliary Cancer Clinic, Department of Surgery, Gangnam Severance Hospital, Yonsei University College of Medicine, Seoul, Korea; 3 Department of Internal Medicine, Gangnam Severance Hospital, Yonsei University College of Medicine, Seoul, Korea; 4 Departments of Radiation Oncology, Gangnam Severance Hospital, Yonsei University College of Medicine, Seoul, Korea

## Abstract

Our aim was to identify the risk factors associated with locoregional recurrence in resected distal bile duct cancer (DBDC), and to determine the subgroup that may benefit from adjuvant radiotherapy. Between 2001 and 2013, we retrospectively analyzed 93 patients with DBDC who had undergone curative resection. Patients who received adjuvant radiotherapy were excluded. The 3-year locoregional failure-free survival (LRFFS) and overall survival (OS) rates for all patients were 50.7%, and 53.2%, respectively. On multivariate analysis, the preoperative carcinoembryonic antigen (CEA) level, resection margin, histologic grade, T stage, and N stage were significant prognostic factors for LRFFS. Locoregional recurrence was observed in more than 78% of the patients who underwent R1 resection and were node-positive, and the 3-year LRFFS rate was 19.3%. The 3-year LRFFS rate was 46.9% in the patients who underwent R0 resection and were node-negative with more than 2 risk factors (preoperative CEA level ≥ 5 ng/mL, poorly differentiated histologic grade, and T3 stage). On multivariate analysis for OS, patients with more than 2 risk factors showed a 7-fold higher risk of death, compared with patients with 1 or no risk factor. The important risk factors of locoregional failure in patients with DBDC who underwent resection were R1 resection and positive lymph nodes. Adjuvant radiotherapy should be considered for these patients to improve the oncologic outcomes. Patients undergoing selective R0 resection and those with node-negative status and multiple locoregional failure risk factors may benefit from adjuvant radiotherapy.

## Introduction

Curative resection is the treatment of choice for distal bile duct cancer (DBDC). However, the prognosis of DBDC remains unsatisfactory even after curative resection, with the 5-year overall survival (OS) rate at only approximately 25–48%^1–7^. One study showed that 52% of patients who underwent resection without adjuvant treatment had disease recurrence^6^. The prognosis after recurrence is dismal, with a median OS of only 6–9 months^1–3^.

Although the efficacy of adjuvant treatment after curative resection remains controversial, adjuvant treatment is commonly used to improve the poor prognosis of DBDC. Adjuvant radiotherapy has been recommended to improve locoregional control and has increased survival^4–6,8–10^.

A better understanding of locoregional recurrence after resection for DBDC is essential in implementing radiotherapy and improving surgical outcomes. However, because of the rarity of DBDC^11,12^, the risk factors for locoregional recurrence have not been well established and research on prognostic factors influencing locoregional failure-free survival (LRFFS) is limited. Therefore, the aim of this study was to focus on locoregional recurrence after curative resection of DBDC. We analyzed the risk factors associated with locoregional recurrence in patients undergoing surgical resection for DBDC and investigated the subgroup of patients who may benefit the most from adjuvant radiotherapy.

## Results

### Patient characteristics

The characteristics of the 93 included patients are listed in Table 1. The median age was 67 years (range, 34–81 years). The median preoperative and postoperative carbohydrate antigen (CA) 19–9 levels were 96.6 U/mL and 14.8 (range, 1.8–4529) U/mL, respectively and the median preoperative carcinoembryonic antigen (CEA) level was 2.5 (range, 0.3–32.1) ng/mL. The preoperative CA 19–9 level of 1 patient was higher than 20040 U/mL. Nineteen patients underwent segmental resection of bile duct; 74 underwent pylorus-preserving pancreatoduodenectomy. After surgical resection, R0 resection was achieved in 79 patients (84.9%), and R1 resection in 14 patients (15.1%). Metastatic lymph nodes (LNs) were found in 39.8% of patients.

**Table 1:** Patient characteristics of all patients and relationship between patient characteristics and loco-regional recurrence.

Variable	Number of patients (%)			
		Loco-regional recurrence		p-value
		Yes	No	
Age (years)				0.277
≤65	35 (40.9)	23 (60.5)	15 (39.5)	
>65	55 (59.1)	27 (49.1)	28 (50.9)	
Sex				0.108
Male	59 (63.4)	28 (47.5)	31 (52.5)	
Female	34 (36.6)	22 (64.7)	12 (35.3)	
Preoperative CA 19–9 (U/mL)				0.011
<37	25 (26.9)	8 (32.0)	17 (68.0)	
≥37	68 (73.1)	42 (61.8)	26 (38.2)	
Postoperative CA 19–9 (U/mL)				0.337
<37	81 (87.1)	42 (51.9)	39 (48.1)	
≥37	12 (12.9)	8 (66.7)	4 (33.3)	
Preoperative CEA (ng/mL)				0.255
<5	73 (78.5)	37 (50.7)	36 (49.3)	
≥5	20 (21.5)	13 (65.0)	7 (35.0)	
Surgical procedure				0.912
PPPD	74 (79.6)	40 (54.1)	34 (45.9)	
Segmental resection of bile duct	19 (20.4)	10 (52.6)	9 (47.4)	
Resection margin				0.009
R0	79 (84.9)	38 (48.1)	41 (51.9)	
R1	14 (15.1)	12 (85.7)	2 (14.3)	
Histologic grade				0.255
WD/MD	73 (78.5)	37 (50.7)	36 (49.3)	
PD	20 (21.5)	13 (65.0)	7 (35.0)	
Lymphovascular invasion				0.161
No	67 (72.0)	33 (49.3)	34 (50.7)	
Yes	26 (28.0)	17 (65.4)	9 (34.6)	
Perineural invasion				0.981
No	28 (30.1)	15 (53.6)	13 (46.4)	
Yes	65 (69.9)	35 (53.8)	30 (46.2)	
T stage (AJCC 7th edition)				0.011
T1-2	41 (44.1)	16 (39.0)	25 (61.0)	
T3	52 (55.9)	34 (65.4)	18 (34.6)	
N stage (AJCC 7th edition)				<0.001
N0	56 (60.2)	21 (37.5)	35 (62.5)	
N1	37 (39.8)	29 (78.4)	8 (21.6)	
N stage (AJCC 8th edition)				0.001
N0	56 (60.2)	21 (37.5)	35 (62.5)	
N1	29 (31.2)	23 (79.3)	6 (20.7)	
N2	8 (8.6)	6 (75.0)	2 (25.0)	
Adjuvant chemotherapy				0.619
No	45 (48.4)	23 (51.1)	22 (48.9)	
Yes	48 (51.6)	27 (56.3)	21 (43.8)	
Locoregional recurrence risk group				<0.001
Group 1	42 (45.2)	34 (81.0)	8 (19.0)	
Group 2	12 (12.9)	6 (50.0)	6 (50.0)	
Group 3	42 (45.2)	10 (25.6)	29 (74.4)	

CA, carbohydrate antigen; CEA, carcinoembryonic antigen; PPPD, pylorus-preserving pancreaticoduodenectomy; WD, well differentiated; MD, moderately differentiated; PD, poorly differentiated; AJCC, American Joint Committee on Cancer

Group 1 was defined as patients with positive resection margin or positive lymph nodes.

Group 2 was defined as patients with negative resection margin and negative lymph nodes who possessed 2 or more risk factors (preoperative CEA level ≥ 5 ng/mL, poorly differentiated histologic grade, and T3 stage).

Group 3 was defined as patients with negative resection margin and negative lymph nodes with 1 or no risk factor (preoperative CEA level ≥ 5 ng/mL, poorly differentiated histologic grade, and T3 stage).

### Survival analysis and patterns of failure

The median follow-up was 46 months (range, 8–155 months) for the surviving patients. Of the surviving patients, 13 (13/45, 26.7%) were followed up for 3 years or less. The median OS was 47 months. The 3-year OS and LRFFS rates were 53.2% and 50.7%, respectively.

During follow-up, 56 patients (60.2%) were diagnosed with recurrence, and locoregional recurrence was the dominant type of failure. Locoregional recurrences were the first event in 50 patients (53.8%), and distant relapse occurred in 40 patients (43.0%). Thirty-four patients (36.6%) had both locoregional relapse and distant metastasis. The liver was the most common site for primary metastatic recurrence.

### Risk factors related to locoregional failure-free survival and overall survival

Initial locoregional recurrence was more frequent in patients with preoperative CA 19–9 level > 37 U/mL, positive resection margin (RM), T3 stage, and positive LNs (Table 1). Initial locoregional recurrence occurred in 85.7% and 78.4% of the patients with positive RM and positive LNs, respectively. Initial locoregional recurrence occurred in 23 (79.3%) of 29 patients who had positive LNs and negative RM. The preoperative and postoperative CA 19–9 level, RM, lymphovascular invasion, T stage, and N stage were significant prognostic factors for LRFFS (p < 0.05) (Table 2). Multivariate analysis showed that preoperative CEA level, RM, histologic grade, T stage, and N stage were independent prognostic factors for LRFFS (p < 0.05) (Table 3).

**Table 2:** Univariate analysis of prognostic factors for locoregional failure-free survival and overall survival.

Prognostic factor	3-y Survival rate (%)			
	LRFFS	p-value	OS	p-value
Age (years)		0.276		0.493
≤65	43.3		48.5	
>65	55.9		56.5	
Sex		0.167		0.636
Male	56.1		60.5	
Female	41.5		39.2	
Preoperative CA 19–9 (U/mL)		0.019		0.014
<37	70.2		78.6	
≥37	43.6		44.6	
Postoperative CA 19–9 (U/mL)		0.001		<0.001
<37	54.4		58.6	
≥37	27.8		16.7	
Preoperative CEA (ng/mL)		0.087		0.056
<5	54.2		57.2	
≥5	37.9		39.4	
Surgical procedure		0.853		0.811
PPPD	50.5		53.5	
Segmental resection of bile duct	51.7		52.1	
Resection margin		<0.001		<0.001
R0	56.5		60.2	
R1	15.9		14.3	
Histologic grade		0.104		0.268
WD/MD	54.1		55.8	
PD	38.2		44.4	
Lymphovascular invasion		0.008		<0.001
No	57.2		64.1	
Yes	33.3		25.2	
Perineural invasion		0.954		0.808
No	47.2		50.8	
Yes	52.2		54.2	
T stage (AJCC 7th edition)		0.003		0.002
T1-2	69.6		69.7	
T3	35.1		40.0	
N stage (AJCC 7th edition)		<0.001		<0.001
N0	70.4		69.8	
N1	19.3		29.0	
N stage (AJCC 8th edition)		<0.001		<0.001
N0	70.4		69.8	
N1	21.6		34.1	
N2	25.0		12.5	
Adjuvant chemotherapy		0.404		0.164
No	58.6		63.0	
Yes	43.1		44.4	
Locoregional recurrence risk group		<0.001		<0.001
Group 1	19.3		28.0	
Group 2	46.9		45.7	
Group 3	84.2		83.5	

CA, carbohydrate antigen; CEA, carcinoembryonic antigen; PPPD, pylorus-preserving pancreaticoduodenectomy; WD, well differentiated; MD, moderately differentiated; PD, poorly differentiated; AJCC, American Joint Committee on Cancer; LRFFS, locoregional failure-free survival; OS, overall survival.

Group 1 was defined as patients with positive resection margin or positive lymph nodes.

Group 2 was defined as patients with negative resection margin and negative lymph nodes who possessed 2 or more risk factors (preoperative CEA level ≥ 5 ng/mL, poorly differentiated histologic grade, and T3 stage).

Group 3 was defined as patients with negative resection margin and negative lymph nodes with 1 or no risk factor (preoperative CEA level ≥ 5 ng/mL, poorly differentiated histologic grade, and T3 stage).

**Table 3:** Multivariate analysis of prognostic factors for locoregional failure-free survival and overall survival.

Variable	LRFFS		OS	
	HR (95% CI)	p-value	HR (95% CI)	p-value
Preoperative CEA (≥5 ng/mL)	2.169 (1.111–4.237)	0.023	—	—
Resection margin (R1)	3.912 (1.835–8.343)	<0.001	—	—
Histologic grade (PD)	2.208 (1.125–4.337)	0.021	—	—
T stage (T3, AJCC 7th edition)	3.473 (1.788–6.747)	<0.001	—	—
N stage (N1, AJCC 7th edition)	4.386 (2.278–8.444)	<0.001	—	—
Locoregional recurrence risk group 3				
vs Group 2	—	—	6.927 (2.120–22.632)	0.001
vs Group 1	—	—	8.258 (3.345–20.388)	<0.001
Preoperative CA 19–9 (≥37 U/mL)	—	—	2.183 (0.926–5.144)	0.074
Postoperative CA 19-9 (≥37 U/mL)	—	—	2.489 (1.199–5.163)	0.014
Lymphovascular invasion (Positive)	—	—	2.339 (1.247–4.387)	0.008

CEA, carcinoembryonic antigen; PD, poorly differentiated; AJCC, American Joint Committee on Cancer; CA, carbohydrate antigen; LRFFS, locoregional failure-free survival; OS, overall survival; HR, hazard ratio; CI, confidence interval.

Group 1 was defined as patients with positive resection margin or positive lymph nodes.

Group 2 was defined as patients with negative resection margin and negative lymph nodes who possessed 2 or more risk factors (preoperative CEA level ≥ 5 ng/mL, poorly differentiated histologic grade, and T3 stage).

Group 3 was defined as patients with negative resection margin and negative lymph nodes with 1 or no risk factor (preoperative CEA level ≥ 5 ng/mL, poorly differentiated histologic grade, and T3 stage).

**Figure 1:**
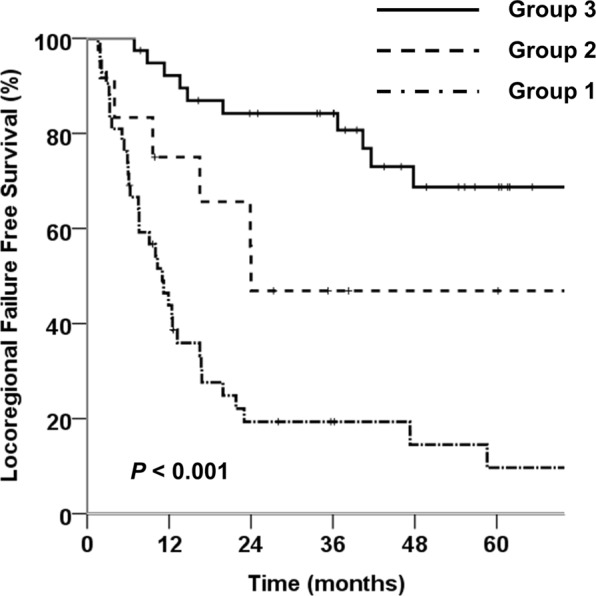
Comparison of locoregional failure-free survival curves according to locoregional recurrence risk groups. Group 1 was defined as patients with positive resection margin or positive lymph nodes. Group 2 was defined as patients with negative resection margin and negative lymph nodes who possessed 2 or more risk factors (preoperative CEA level ≥ 5 ng/mL, poorly differentiated histologic grade, and T3 stage). Group 3 was defined as patients with negative resection margin and negative lymph nodes with 1 or no risk factor (preoperative CEA level ≥ 5 ng/mL, poorly differentiated histologic grade, and T3 stage).

All patients were divided into 3 locoregional recurrence risk groups according to risk factors (Table 4). As locoregional recurrence occurred in more than 78% of the patients with positive RM and LNs, these patients were defined as the high-risk group for locoregional recurrence (group 1, n = 42). The patients, with negative RM and LNs, were divided into 2 groups according to the preoperative CEA level, histologic grade, and T stage considering the 5 independent prognostic factors for LRFFS on multivariate analysis. The patients, who had more than 2 risk factors considering preoperative CEA level ≥ 5 ng/mL, poorly differentiated histologic grade, and T3 stage, were defined as group 2 (n = 12), and those who had less than 1 risk factor were defined as group 3 (n = 39). The locoregional recurrence rates of groups 1, 2, and 3 were 81.0%, 50.0%, and 25.6%, respectively (Table 1). The 3-year LRFFS for groups 1, 2, and 3 were 19.3%, 46.9%, and 84.2%, respectively (Fig. 1). LRFFS was significantly different among the three groups (p < 0.05) (Table 2). Moreover, there were significant differences in LRFFS between groups 1 and 2, groups 2 and 3, and groups 1 and 3 (p < 0.05).

**Table 4:** Locoregional recurrence risk groups according to locoregional recurrence risk factors.

Group	Definition	Number of Patients
Group1	Patients with R1 resection or positive lymph node	42
Group2	R0 resection and N0 patients who possessed two to three risk factors (preoperative CEA level ≥ 5 [ng/mL], poorly differentiated histologic grade, and T3 stage)	12
Group3	R0 resection and N0 patients who did not exhibit any risk factors or had one risk factor (preoperative CEA level ≥ 5 [ng/mL], poorly differentiated histologic grade, and T3 stage)	39

CEA, carcinoembryonic a ntigen.

The results of univariate analysis of OS are summarized in Table 2. Univariate analysis showed that preoperative and postoperative CA 19–9 levels, RM, lymphovascular invasion, T stage, N stage, and locoregional recurrence risk group were significantly associated with OS (p < 0.05). For multivariate analysis, the analysis was conducted by substituting locoregional recurrence risk group. On multivariate analysis, locoregional recurrence risk group, postoperative CA19-9 level, and lymphovascular invasion were significantly associated with OS (P < 0.05) (Table 3). The patients in groups 1 and 2 showed approximately 8-fold and 7-fold increased risks of death, respectively, as compared with group 3 (p < 0.05).

## Discussion

The role of adjuvant radiotherapy has not been well established. The literature regarding the benefit of adjuvant radiotherapy for DBDC is inconsistent^4–6,8,9,13^. For the appropriate selection of radiotherapy, risk factors associated with locoregional failure are thought to be an integral part of DBDC management. Several studies reported that 47–66% of patients with DBDC still experienced disease recurrence after undergoing surgical resection, and the initial locoregional recurrence rate was 26–65%^1–3,6,7,14,15^. This study was conducted in patients with DBDC who underwent resection but did not receive adjuvant radiotherapy, and the initial locoregional recurrence rate was 53.8%. Accordingly, adjuvant radiotherapy should be administered for selected patients with DBDC who undergo resection and are at a higher risk of locoregional failure regardless of whether the dominant type of failure is a locoregional recurrence or distant metastasis.

In this study, RM and LNs were found to be high risk factors of locoregional recurrence. However, Kim *et al*. found that N stage and perineural invasion were significantly associated with locoregional control in patients with resected extrahepatic biliary tract cancer^16^, while Choi *et al*. reported that the LN status and RM status were not significant factors for locoregional recurrence, and that poorly differentiated histologic grade was an independent prognostic factor for locoregional recurrence^1^. The discrepancy in the risk factors among the studies can be partly explained by the fact that radiotherapy is usually performed in patients with positive RM. In other words, as adjuvant radiotherapy for patients with positive RM might be effective in controlling microscopic residual tumors, the RM may not be a risk factor of locoregional recurrence. A recent meta-analysis demonstrated that adjuvant chemotherapy or chemoradiotherapy significantly improved the survival of patients with biliary tract cancer with positive LNs or positive RM^9^. Our previous study showed that adjuvant chemoradiotherapy improved the clinical outcomes of patients with resected extrahepatic bile duct cancer, particularly patients who underwent R1 resection^8^. Kim *et al*. reported that adjuvant chemotherapy or chemoradiotherapy was associated with a significant OS advantage in patients with LN-positive R0-resected DBDC^6^. Therefore, adjuvant radiotherapy should be considered for locoregional control of RM-positive or LN-positive DBDC.

The benefit of adjuvant radiotherapy remains unclear for patients with DBDC and negative LNs treated with R0 resection. In this study, the locoregional recurrences rate was 25.6% in patients with 1 or no risk factor among preoperative CEA level ≥ 5 ng/mL, poorly differentiated histologic grade, and T3 stage. Therefore, we believe that adjuvant radiotherapy may not improve the clinical outcomes of these patients. However, locoregional recurrence occurred in 50.0% of the patients who underwent R0 resection and had negative LNs with more than 2 risk factors, and the 5-year LRFFS was 46.9%; thus, patients with more than 2 risk factors showed a 7-fold higher risk for death compared with those with 1 or no risk factors on multivariate analysis for OS. Adjuvant radiotherapy may be helpful to increase survival outcome for some of these patients. Nevertheless, further studies should be performed to determine whether adjuvant radiotherapy can actually improve clinical outcomes of these patients.

There are several limitations of this study. First, this was a nonrandomized and retrospective study, and unrecognized bias could be present. As this study was conducted at a single center, and the patients treated with adjuvant radiotherapy were excluded, selection bias may have affected the results. Further, 13 surviving patients were followed up for 3 years or less which may also have generated selection bias. Additionally, because the present study involved a limited number of patients, the results of this study must be confirmed in larger studies in the future. Finally, CA 19–9 and CEA levels could show a false positive or negative result. Therefore, caution is needed when evaluating the recurrence or prognosis of the patients^17^.

In conclusion, R1 resection and positive LNs were important prognostic factors for locoregional recurrence in patients with DBDC. Therefore, we recommend adjuvant radiotherapy with curative resection for patients with DBDC treated with R1 resection or those who have positive LNs. Adjuvant radiotherapy for patients treated with R0 resection and those with negative LNs who exhibit multiple locoregional risk factors may reduce locoregional failure, which translates into OS benefits. Future well-designed, randomized, controlled studies are necessary to clarify the role of adjuvant radiotherapy.

## Methods

The study protocol conformed to the ethical guidelines of the 1975 Declaration of Helsinki, as revised in 1983, and was approved by Institutional Review Board of Severance Hospital. The patient records/information was anonymized and de-identified prior to analysis, and informed consent was not obtained from each participant. After Institutional Review Board approval, we retrospectively reviewed the records of all patients with DBDC adenocarcinoma who underwent curative surgical resection between January 2001 and December 2013. The inclusion criteria were no distant metastasis and an Eastern Cooperative Oncology Group performance status of ≤2. Patients were excluded if they received neoadjuvant therapy or adjuvant radiotherapy; patients with ampullary and duodenal carcinomas were also excluded from this study. Finally, 93 patients with DBDC, confirmed by pathological diagnosis after surgical resection, were included in this study. The primary outcome was the 3-year LRFFS in patients undergoing surgical resection for distal bile duct cancer; the secondary outcomes were OS and patterns of failure.

The type of surgery selected was determined according to the location of the tumor: if the tumor was located in the distal bile duct, pancreaticoduodenectomy was performed; if the tumor was located in the mid portion of the bile duct, segmental resection and hepaticojejunostomy were performed. All patients underwent dissection of the LNs in the hepatoduodenal ligament, as well as common hepatic artery and celiac axis LNs. Patients with histologically positive LNs or poorly differentiated DBDC considering the histologic grade were usually treated with adjuvant chemotherapy. Adjuvant 5-fluorouracil-based or gemcitabine-based chemotherapy was administered at the physician’s discretion.

The disease stage for all patients was determined according to the 7th edition of the American Joint Committee on Cancer System. Positive margins were defined as the presence of at least 1 cancer cell at the RM on microscopic examination. CA 19–9 level of 37 U/mL and CEA level of 5 ng/mL was defined as a cutoff value for normal level^2,6,8,15–17^.

Patients were followed-up 1 month after the operation and every 3 months during the first 12 months, and then every 6 months beyond the first year. Patients underwent measurements of CA 19–9 and CEA, and underwent computed tomography. The postoperative CA 19–9 level was measured between 1 week and 1 month after surgical resection. Tumor recurrences were confirmed using radiological imaging techniques and pathological studies of histological specimens, with the majority being made by radiological evaluation, including computed tomography and positron emission tomography. Recurrence was also confirmed pathologically by biopsy or via radiological findings. In this study, only the initial recurrence was evaluated. Locoregional recurrence was defined as recurrence in the primary tumor bed and regional lymphatic areas. All other recurrences were considered distant metastasis.

Survival was calculated from the date of surgical resection. All events were measured from the date of surgery to the date of recurrence. A chi-square test or Fisher exact test was used for comparison of categorical variables between groups. Survival was estimated by using the Kaplan–Meier method and compared with the log-rank test. Multivariate analysis was performed using a Cox proportional hazards model, and the hazard ratio with a 95% confidence interval was determined. We performed a multivariate analysis using backwards elimination to stay in the model. A p-value of <0.05 indicated statistical significance.
